# Advanced heart disease classification based on multi-channel heart sound coupling features

**DOI:** 10.1371/journal.pone.0321209

**Published:** 2025-05-23

**Authors:** Yu Fang, Dongbo Liu, Zijian Guo, Hongxia Leng, Xing Liu, Xiaochen Wu

**Affiliations:** 1 School of Electrical Engineering and Electronic Information, Xihua University, Chengdu, China; 2 Department of Cardiovascular, General Hospital of Western Command Theater, Chengdu, China; University of Manitoba, CANADA

## Abstract

Conventional heart sound classification methods often rely on single-channel, one-dimensional feature extraction, which inadequately captures pathological relationships across different auscultation zones, thereby limiting the accuracy of heart disease detection. To address this issue, a novel classification framework based on multi-channel heart sound coupling feature extraction is proposed to enhance heart disease identification. This approach begins with denoising preprocessing applied to four-channel heart sound signals and a single-channel electrocardiogram. These five-channel signals are systematically paired to extract five types of coupling features, resulting in 130 distinct features per multi-channel sample. The ReliefF algorithm is then used to evaluate feature importance, retaining the top 20% of features to construct a coupling feature set. A convolutional neural network is employed to classify normal and abnormal heart sounds. When applied to clinical congenital heart disease datasets, the proposed method achieved a classification accuracy of 95.6%, while on the PhysioNet heart sound challenge dataset, it reached an accuracy of 98.3%. Experimental results demonstrate that compared to single-channel, one-dimensional features, multi-channel coupling features more effectively capture pathological characteristics in heart sound signals, significantly improving the accuracy of heart disease classification and addressing challenges in the refined categorization of cardiac conditions.

## Introduction

Cardiovascular diseases continue to be among the leading causes of mortality worldwide [1]. Although many cardiac conditions can be managed through pharmacological treatments or surgical interventions, the early detection and monitoring of congenital heart disease (CHD) remain critical. Without timely intervention, CHD can result in significant long-term health complications, such as heart failure, pulmonary hypertension, and life-threatening arrhythmias. Therefore, regular medical follow-ups and continuous monitoring are essential for CHD patients.

Heart sounds, produced by the movement of blood during the cardiac cycle, mainly comprise two key components: the first heart sound (S1) and the second heart sound (S2). These sounds correspond to the transitions between diastole and systole, and the beginning of diastole following systole, respectively [2]. In addition to these core sounds, murmurs may arise from abnormalities, including valvular dysfunction, valve malformation, myocardial hypertrophy, or ischemia, signaling potential cardiac issues [3]. By analyzing the characteristics of heart sounds—such as the presence of murmurs, their timing, intensity, and quality—clinicians can assess heart health and identify possible diseases. Variations in sound intensity or frequency often reflect changes in myocardial contractility or valvular function, enabling initial evaluations through auscultation [4].

During clinical evaluations, several auscultation sites are examined, including the aortic, pulmonary, tricuspid, and mitral valve areas [5]. Given the anatomical and hemodynamic properties of the heart, interactions between different auscultation sites can yield valuable diagnostic information. Analyzing multi-channel signals provides a more comprehensive evaluation, improving diagnostic accuracy.

Feature extraction and classifier selection are critical elements of heart sound classification. Recent studies have introduced various techniques: Nizam NB et al. extracted Hilbert envelope features and cardiac cycle features from preprocessed heart sound signals and employed a random forest classifier to analyze the Yaseen database, achieving an average classification accuracy of 94.78% [6]. Deng et al. proposed a classification method based on improved Mel-frequency cepstral coefficients (MFCC) and convolutional recurrent neural networks, achieving a classification accuracy of 98% for distinguishing pathological from non-pathological heart sounds [7]. Deperlioglu et al. employed signal energy features and stacked autoencoders, achieving a 99.61% classification accuracy on the PASCAL-B dataset [8]. Singh et al. used wavelet decomposition, homomorphic filtering, and power spectral density to extract coupled features from Electrocardiogram (ECG) and Phonocardiogram (PCG), achieving 93.13% accuracy in the PhysioNet/CinC 2016 challenge [9]. Choudhary et al. performed a five-level signal decomposition using discrete wavelet decomposition and conducted binary classification on the PhysioNet/CinC 2016 dataset by utilizing convolutional neural networks and GRU network layers [10]. Liu et al. introduced a heart sound classification method combining bispectrum features and the Vision Transformer model, using data from the PhysioNet Challenge 2016 and 2022 databases to enhance early cardiovascular disease diagnosis [11]. While many studies examine single-channel signals using one-dimensional feature analysis, this approach often overlooks crucial information present in multi-channel data. Specifically, the focus on one-dimensional, chain-like features in single channels neglects the richness of multi-channel signals, potentially leading to a loss of vital internal information.

Several studies have investigated various features of heart sound signals for cardiac disease diagnosis, including bispectral, homomorphic, Hilbert, power spectral density, wavelet envelope, and traditional MFCC features. Zeye Liu et al. proposed a classification algorithm using bispectral features and Vision Transformer models, achieving 91% accuracy on the PhysioNet Challenge 2022 dataset [12]. Sofia Monteiro et al. combined homomorphic, Hilbert, and wavelet envelope features with BiLSTM networks, achieving 75.1% accuracy [13]. Yunendah Nur Fuadah et al. applied MFCC features with various classifiers, achieving 76.31% accuracy on the 2022 heart sound dataset [14]. However, a common limitation is that these studies, despite utilizing multi-channel signals, neglect the inter-channel relationships and lack analysis of the coupling between them. Consequently, their classification performance remains suboptimal. Ali et al. proposed a novel methodology leveraging a hybrid approach that combines energy and entropy features to identify distinct characteristics from one-dimensional heart sound signals [15]. Liu et al. collected 5-minute multi-channel heart sound signals from 21 CAD patients and 15 non-CAD subjects, extracting time-domain, frequency-domain, entropy, and cross-entropy features, with a support vector machine achieving 90.92% classification accuracy [16]. Samanta et al. collected heart sound signals from four chest auscultation sites in CAD patients and normal subjects, extracting features such as approximate entropy, maximum Lyapunov exponent, mutual information, and path length entropy, resulting in a classification accuracy of 82.57% using an artificial neural network [17].While these studies analyze multi-channel heart sound datasets, they often fail to consider the coupling relationships between channels, leading to suboptimal classification performance.

Research indicates that multi-channel heart sound signals provide richer pathological information, enhancing classification performance. However, the majority of existing studies fail to fully exploit this potential by neglecting the crucial inter-channel relationships within heart sounds. Recognizing that understanding these inter-channel connections is vital for improving diagnostic accuracy and care for cardiovascular patients, this paper introduces a novel heart sound classification method. In this proposed approach, multi-channel coupling features are leveraged, and machine learning models are employed for classification. The experimental results demonstrate that a significant enhancement in classification accuracy is achieved by this method, offering clinicians a more precise and efficient diagnostic tool for cardiac diseases.

## Multi-channel heart sound coupling feature extraction

The multi-channel heart sound dataset utilized in this study comprises 293 heart sound samples, including 126 normal heart sounds and 167 abnormal heart sounds. The dataset is primarily sourced from two parts. First, a cardiovascular surgery dataset from the department of cardiovascular surgery general hospital of the western theater command people’s liberation army of china comprising 126 heart sound samples was utilized, including 18 normal heart sounds and 108 abnormal heart sounds. Second, 167 heart sound samples were selected from the PhysioNet 2022 heart sound challenge dataset, consisting of 108 normal heart sounds and 59 abnormal heart sounds.

**Table 1:** Summary of the PhysioNet 2022 heart sound challenge dataset and clinical dataset.

Dataset	Sample Category	Number of Samples
2022 Heart Sound Challenge Dataset	Normal	108
	Abnormal	59
	Total	167
Clinical Dataset	Normal Heart Sounds	18
	Atrial Septal Defect	33
	Ventricular Septal Defect	51
	Patent Ductus Arteriosus	6
	Tetralogy of Fallot	16
	Total	126

This study was performed in line with the principles of the Declaration of Helsinki. Approval was granted by the ethics committee of General Hospital of Western Command Theater (No. 2015 research 01).

Regarding data acquisition, the signals in the cardiovascular surgery dataset are measured in volts (Volts), with a sampling rate of 20 kHz. This dataset encompasses four-channel heart sound signals from the aortic valve (A), pulmonary valve (P), tricuspid valve (T), and mitral valve (M), along with one ECG signal. The Challenge 2022 dataset also contains the same four-channel signals, but with a sampling frequency of 4 kHz.

**Fig 1:**
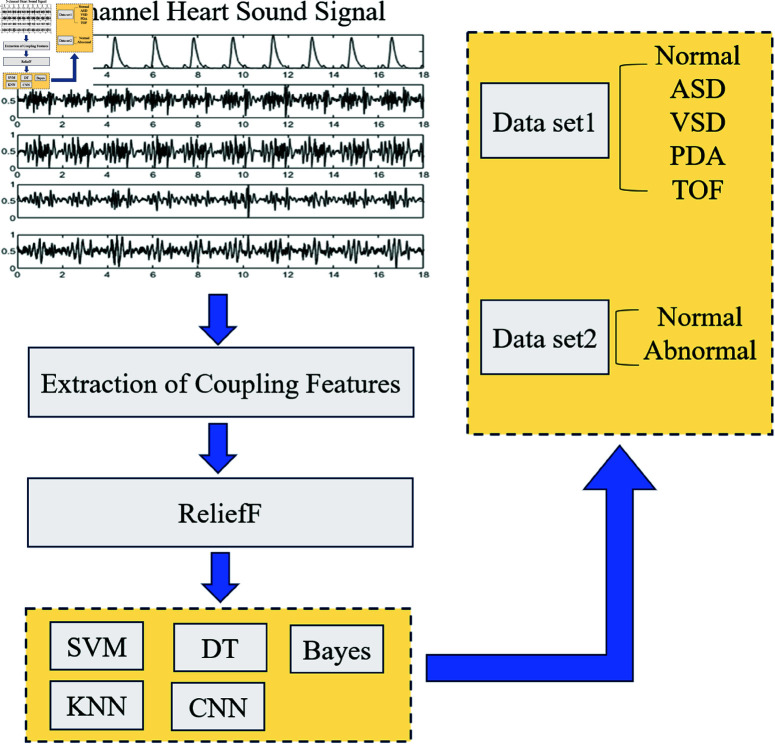
Overall flowchart of the algorithm.

The proposed algorithm consists of four steps: preprocessing, coupling feature extraction, dimensionality reduction using the ReliefF method, and classification. The overall workflow of the algorithm is illustrated in Fig 1.

Initially, a Butterworth filter is employed to denoise the multi-channel heart sound signals. The filter cutoff frequency is set between 4.85 Hz and 1250 Hz, allowing the retention of effective heart sound information while removing noise or interference within specific frequency ranges [18,19].

**Fig 2:**
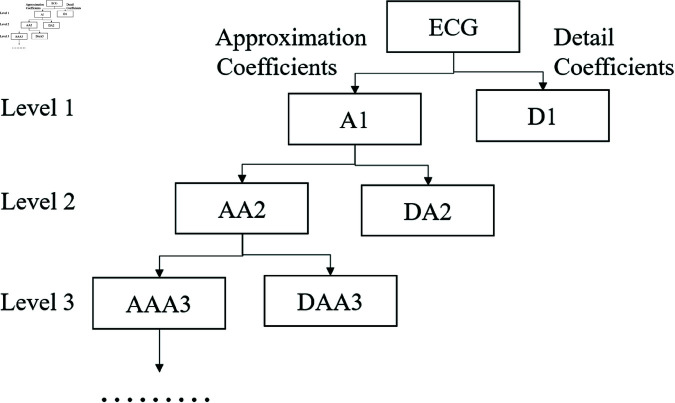
Diagram of wavelet decomposition.

Subsequently, a discrete wavelet transform algorithm [20] is applied to detect and remove the trend components from the ECG signal. The Daubechies wavelet is selected, and the original ECG signal undergoes a ten-level decomposition. To eliminate long-term trends, the approximate coefficients of the tenth level are discarded. The ECG signal is denoted as s(n), where n=1,2,3,…,N, with *N* representing the signal duration. The formula for the discrete wavelet transform is expressed as follows:

$$W_{j,k}=\sum_{n=1}^{N}s(n)\cdot \psi_{j,k}(n)$$ (1)

where *W*_*j*,*k*_ represents the wavelet coefficient at level *j* and position *k*, and $\psi_{j,k}(n)$ is the translated discrete wavelet basis function. The filter coefficient sequence associated with the wavelet decomposition is denoted as h(n), and *n* indicates the sample points of the signal. Fig 2 illustrates the spatial partitioning diagram of wavelet decomposition, demonstrating the three-level decomposition process, which can be similarly extended for multiple levels. The original ECG signal is decomposed into high-frequency (D) and low-frequency (A) components, with the numerical values indicating the level of decomposition.

**Fig 3:**
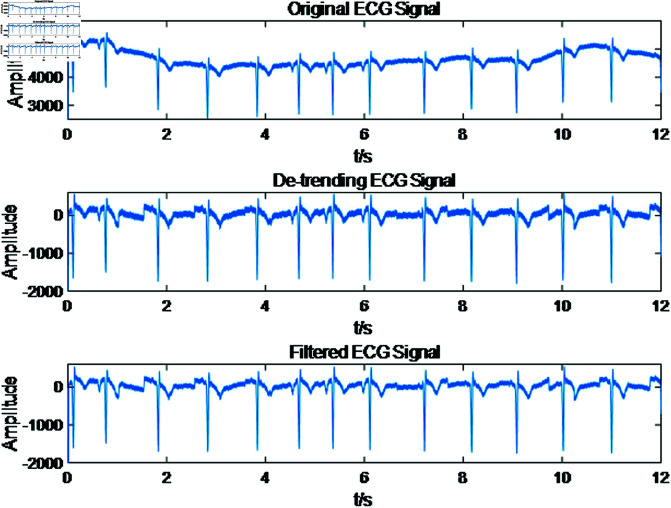
Electrocardiogram signal preprocessing diagram.

To eliminate noise interference from the ECG signal, a Butterworth bandpass filter with cutoff frequencies of 5 Hz and 15 Hz is utilized. The de-trended electrocardiogram and the filtered ECG signals are depicted in Fig 3. Compared to the original ECG signal, the processed signal exhibits a smoother waveform, with the R-peaks and other key features becoming more pronounced and clearly visible.

**Fig 4:**
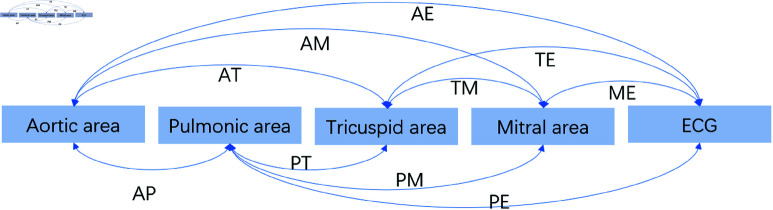
Multichannel heart sound coupling combinations diagram.

The clinical dataset used in this study comprises four channels of heart sound signals corresponding to the auscultation areas of the aortic valve (A), pulmonary valve (P), tricuspid valve (T), and mitral valve (M), along with one channel of ECG signal (denoted as E). By extracting coupling features from different pairs of channels, a total of 10 combination forms are obtained: AP, AT, AM, AE, PT, PM, PE, TM, TE, and ME. Since coupling features are extracted from every pair of signals among the five channels, the number of combinations is calculated using the combination formula (52)=10. The combination forms are illustrated in Fig 4. For each combination, 13 coupling features are extracted. Each coupling method is applied separately to the multi-channel dataset, resulting in a total of 130 coupling features.

### Mutual entropy analysis

Mutual Sample Entropy (SamEn) is a technique used to analyze the asynchrony between two correlated time series [21]. The more regular the coupling pattern between the two sequences, the lower the value of mutual sample entropy, and conversely, the more complex the pattern, the higher the entropy value. It extends the design concept of sample entropy but focuses on comparing sequence segments from one time series with corresponding segments in another, thereby measuring the level of synchrony or consistency between the two time series. Mutual sample entropy evaluates the temporal dynamic characteristics of two signals by calculating the probability that paired segments from the two normalized heart sound channels, denoted as x(i) and y(j), remain similar under a given distance threshold.

Let X(i) and Y(j) represent the reconstructed state space of two signals:


X(i)=[x(i),x(i+1),…,x(i+m−1)],1≤i≤N−m+1

Y(j)=[y(j),y(j+1),…,y(j+m−1)],1≤j≤N−m+1

where *m* is the embedding dimension, and *r* is the threshold parameter. The calculation steps for mutual sample entropy are as follows:

First, the reconstructed state space is used to compute:


$$B_{i}^{m}(r)=\frac{1}{N-m+1}\sum_{j=1,j\neq i}^{N-m+1}A(d^{m}(X(i),Y(j))-r)$$

where A(−) is the Heaviside function, and *d*^*m*^ represents the distance between two segments. The average value of $B_{i}^{m}(r)$ is computed as:


$$B^{m}(r)=\frac{1}{N-m+1}\sum_{i=1}^{N-m+1}B_{i}^{m}(r)$$

The mutual sample entropy is then given by:


$$SamEn(m,r,N)=-\ln\left(\frac{B^{m+1}(r)}{B^{m}(r)}\right)$$

**Fig 5:**
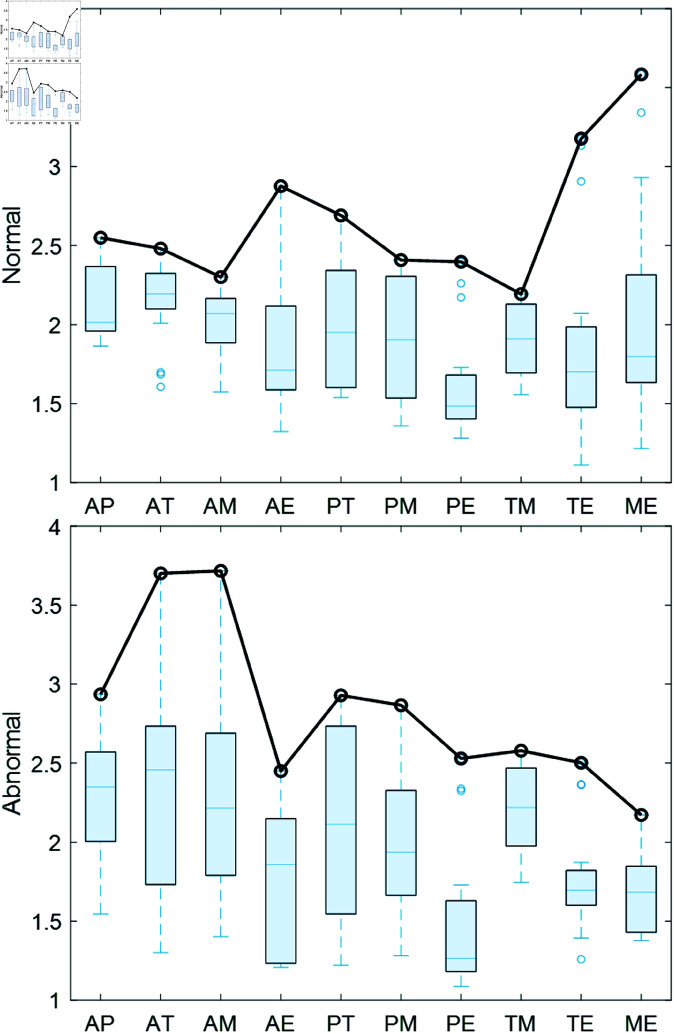
Box plots of mutual sample entropy for heart sound signals.

Fig 5 shows the box plots of mutual sample entropy for all channel combinations of normal and abnormal heart sound signals in the clinical dataset. The distribution of mutual sample entropy for normal heart sound signals is more concentrated compared to that of abnormal heart sound signals. As illustrated in Fig 5, there are significant differences in the overall coupling trends between normal and abnormal features, particularly in the first three and last three values along the x-axis.

Mutual Fuzzy Entropy (FuzEn) is often used to assess the complexity of time series [22]. It measures the synchrony or similarity between different signals and, compared to sample entropy, is more effective for handling nonlinear, non-Gaussian, and short data records. By applying fuzzy set theory to similarity functions, mutual fuzzy entropy is less sensitive to noise, thereby enhancing robustness. The primary difference from mutual sample entropy lies in the use of a Gaussian function instead of the Heaviside function, reducing the strict requirement on the threshold *r*. The mutual fuzzy entropy is calculated as:


$$B^{m}(r)=\frac{1}{N}\sum_{i,j=1j\neq i}^{N}\exp\left(-\frac{d_{ij}^{m}}{r}\right)$$

where *m* is the embedding dimension and *r* is the threshold parameter.

Joint Distribution Entropy (JDistEn) is a measure used to quantify the uncertainty in the joint distribution of multidimensional random variables, revealing the complex dependencies between multichannel data [23]. This fundamentally addresses the entropy dependence on *r*, providing a comprehensive measure of coupling between channels. JDistEn is defined based on the empirical probability density function approximated by a histogram with *B* bins:


$$JDistEn(m,B,p)=-\sum_{t=1}^{B}p_{t}\log_{2}(p_{t})$$

where *p*_*t*_ represents the probability of each bin *t*.

### Phase locking value

Phase Locking Value (PLV) is a commonly used metric for quantifying the phase synchrony between two signals at a specific frequency. It reflects whether the instantaneous phases of two signals remain consistent over a specific time period or within a series of time windows. PLV is widely applied in fields such as neuroscience, ECG analysis, and other domains to study the dynamic interactions between physiological signals. The phase of a signal typically represents a specific part of its periodic variation, such as the rising edge, peak, or other significant points of a sine wave. If two periodic signals are aligned at the same point in each cycle, they are considered phase-locked.

The calculation of PLV between signals x(i) and y(j) involves several steps:

First, the analytic signals of x(i) and y(j) are obtained via the Hilbert transform:


$$x_{ana}(i)=x(i)+jH\{x(i)\},\;y_{ana}(j)=y(j)+jH\{y(j)\}$$

where *H* represents the Hilbert transform operation that generates a signal orthogonal to the original signal.

The instantaneous phases are then extracted:


$$\phi_{x}(i)=\arg(x_{ana}(i)),\;\phi_{y}(j)=\arg(y_{ana}(j))$$

The phase difference is computed for each time point i,j:


$$\Delta \phi_{xy}(ij)=\phi_{x}(i)-\phi_{y}(j)$$

Finally, the PLV is calculated by averaging the phase differences over all time points:


$$PLV_{xy}=\frac{1}{N}\left|\sum_{k=1}^{N}e^{j\Delta \phi_{xy}(k)}\right|$$

**Fig 6:**
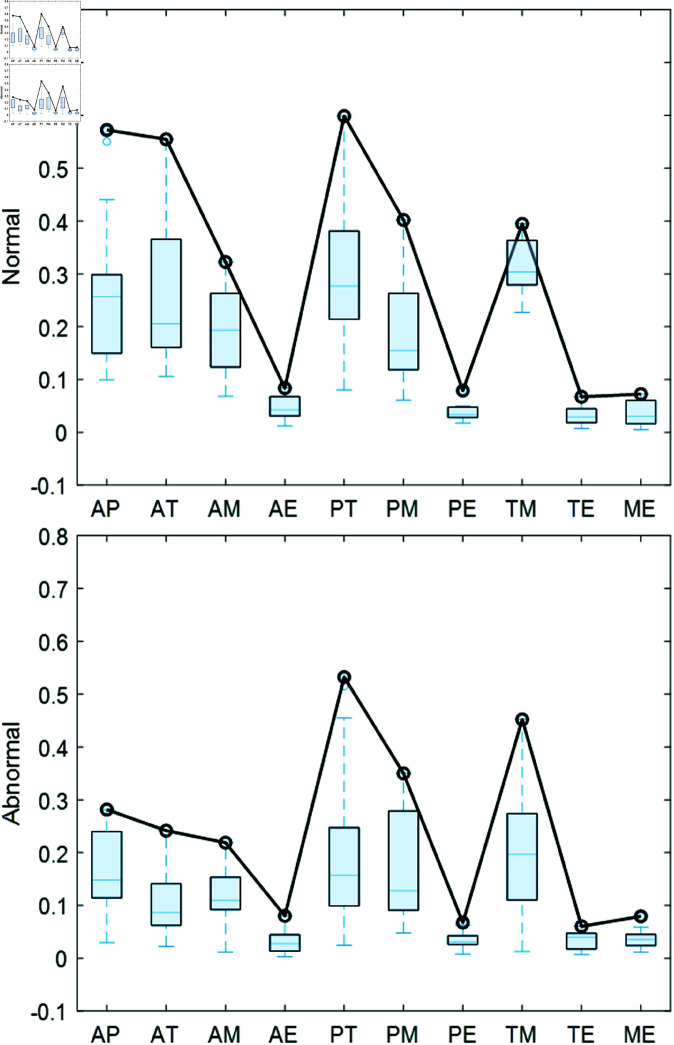
Box plots of PLV values for heart sound signals.

The PLV ranges from 0 to 1, where a value close to 1 indicates strong phase consistency between the two signals, while a value close to 0 suggests little or no phase synchrony. Fig 6 illustrates the PLV values for different channel combinations of normal and abnormal heart sound signals from the clinical dataset. As shown in Fig 6, although the trends of the PLV values for normal and abnormal heart sound types are quite similar, the PLV values for normal heart sounds are more spread out across the first three coordinates on the x-axis, while the values for abnormal heart sounds are concentrated within the 0.1-0.25 range.

### Magnitude squared coherence

Magnitude Squared Coherence (MSC) is a frequency-domain statistic used to evaluate the linear correlation between two signals in terms of their frequency components. It quantifies the strength or consistency of synchronized variations across frequencies between two heart sound channels. The MSC is calculated using the following formula:


$$C_{xy}^{2}(f)=\frac{|P_{xy}(f){|}^{2}}{P_{xx}(f)P_{yy}(f)}$$

where *P*_*xx*_(*f*) and *P*_*yy*_(*f*) are the power spectral densities of x(i) and y(j), respectively, and *P*_*xy*_(*f*) is the cross-power spectral density of x(i) and y(j). In this study, the mean and standard deviation of MSC are extracted as coupling features for further analysis.

### Waveform similarity measures

Waveform similarity measures are metrics used to assess the consistency or similarity between the shapes and patterns of multichannel signals. Heart sound signals contain fundamental information about cardiac activity, and similar patterns are produced in different channels due to the closing and opening of heart valves. Therefore, assessing the waveform similarity quality of multichannel heart sound signals provides valuable insights into the interactions between different regions of the heart. Several metrics are used to quantify waveform similarity, which can reflect coupling strength, temporal relationships, and spatial consistency, providing potential value for heart disease monitoring.

The correlation coefficient [24] is used to quantify the linear dependence between two heart sound signals. When two heart sound signals from different locations are correlated, they may exhibit similar patterns and fluctuations over a complete cardiac cycle. A correlation coefficient close to +1 indicates a positive correlation, meaning that the peaks and troughs of the two signals are synchronized; a value close to -1 indicates a negative correlation, where the peaks and troughs vary in opposite directions; and a value of 0 indicates no linear relationship. The formula for calculating the correlation coefficient is as follows:


$$R_{xy}=\frac{\sum_{k=1}^{N}(x(k)-\bar{x})(y(k)-\bar{y})}{\sqrt{\sum_{k=1}^{N}(x(k)-\bar{x}{)}^{2}\sum_{k=1}^{N}(y(k)-\bar{y}{)}^{2}}}$$

where $\bar{x}$ and $\bar{y}$ represent the mean values of x(i) and y(j), respectively, and *N* is the number of sampling points.

Cosine similarity (CosSim) [25] measures the angle between two vectors representing multichannel heart sound signals, assessing the degree of difference between them. When two heart sound signals are completely identical, their cosine similarity value is maximized (equal to 1). This measure is less sensitive to the length and amplitude of the signals, primarily focusing on whether the waveforms’ patterns and structures match. The formula for cosine similarity is given by:


$$\text{CosSim}(x,y)=\frac{\sum_{k=1}^{N}x(k)y(k)}{\sqrt{\sum_{k=1}^{N}x(k{)}^{2}\sum_{k=1}^{N}y(k{)}^{2}}}$$

Euclidean distance provides an intuitive measure of point-to-point differences between two heart sound signals. Simply put, it measures the root of the sum of squared differences between the amplitudes of the heart sound signals at corresponding time points. A smaller Euclidean distance indicates greater similarity between the waveforms of the two signals. The formula for Euclidean distance is:


$$D_{xy}=\sqrt{\sum_{k=1}^{N}(x(k)-y(k){)}^{2}}$$

### Cross power spectral density

Cross Power Spectral Density (CPSD) is an important tool for analyzing and characterizing the frequency-domain dependencies between two signals. It describes how one signal varies as a function of another signal at a given frequency. CPSD is the Fourier transform of the cross-correlation function between the two signals and is defined by the following formula:


$$S_{XY}(f)=\frac{1}{M}\sum_{m=1}^{M}X_{m}(f)Y_{m}^{*}(f)$$

where * denotes the complex conjugate, and *X*_*m*_(*f*) and *Y*_*m*_(*f*) are the discrete Fourier transforms of the *m*-th windowed segments of signals x(i) and y(j), respectively. *M* represents the number of windows. In this study, the mean and standard deviation of the real and imaginary parts of CPSD are extracted as coupling features for further analysis.

## Feature analysis

**Fig 7:**
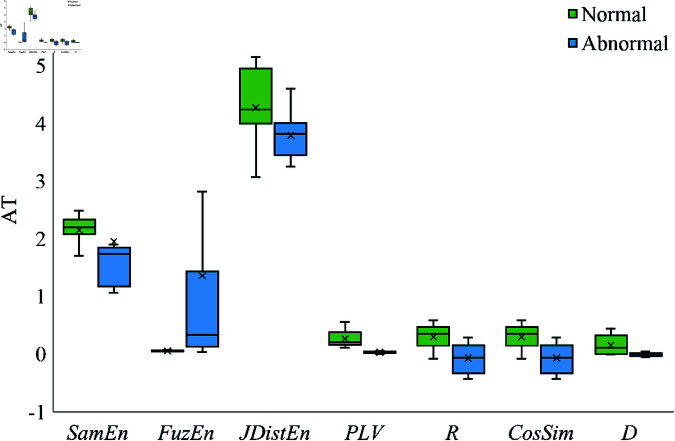
Box plots of coupling features between normal and abnormal signals on channels A and T.

**Fig 8:**
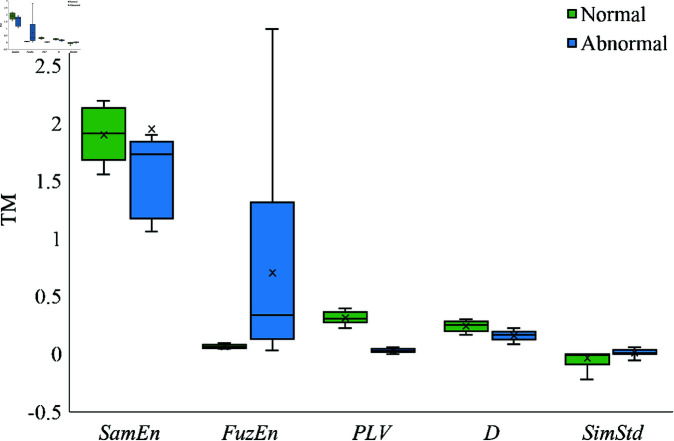
Box plots of coupling features between normal and abnormal signals on channels T and M.

To evaluate the effectiveness of the extracted coupling features in distinguishing between normal and abnormal heart sound signals, a comprehensive analysis was conducted. Figs 7 and 8 present box plots of selected coupling features for the channel pairs A-T and T-M, respectively. These features include mutual sample entropy, phase locking value, correlation coefficient, and magnitude-squared coherence, representing different aspects of the inter-channel relationships.

In Fig 7, it can be observed that the mutual sample entropy between channels A and T shows a noticeable difference between normal and abnormal signals. Specifically, abnormal heart sounds tend to exhibit lower entropy values compared to normal heart sounds. This suggests that pathological conditions may lead to a reduction in the complexity of the coupling patterns between these channels. The decreased entropy indicates more regular or predictable interactions, which could be associated with the presence of cardiac anomalies affecting the synchronization of heart sounds.

The phase locking value (PLV) between channels A and T is generally higher in abnormal signals. A higher PLV indicates stronger phase synchronization, implying that the timing of heart sound events in these channels is more consistently aligned in pathological cases. This could be due to structural or functional abnormalities in the heart that cause simultaneous alterations in the signals recorded from these auscultation sites.

Similarly, the correlation coefficient shows an increased value for abnormal heart sounds between channels A and T. This heightened linear relationship suggests that abnormal conditions may cause the signals in these channels to vary in a more similar fashion, potentially due to shared pathological influences affecting both areas of the heart.

The magnitude-squared coherence displays higher coherence levels in abnormal heart sounds across certain frequency bands. This implies that there is greater consistency in the frequency components of the signals between channels A and T when a heart disease is present. Such findings may be indicative of abnormal mechanical vibrations or turbulent blood flow patterns resulting from cardiac defects.

Fig 8 illustrates the coupling features between channels T and M. The mutual fuzzy entropy is observed to be lower in abnormal heart sounds. This further supports the notion that pathological heart conditions reduce the complexity and increase the regularity of inter-channel interactions. The cosine similarity between channels T and M is higher for abnormal signals, indicating greater waveform similarity. This suggests that abnormal heart conditions may cause similar alterations in the signal shapes at these two locations.

The Euclidean distance between channels T and M is generally smaller for abnormal heart sounds. A reduced Euclidean distance indicates that the amplitude differences between the signals are less pronounced, which could be due to the homogenizing effect of certain heart diseases on the mechanical activities recorded at these sites.

These observations collectively suggest that abnormal heart sounds tend to exhibit more synchronized, less complex, and more similar inter-channel relationships compared to normal heart sounds. The pathological changes in the heart may lead to alterations in the mechanical and electrical activities that are reflected in the coupling features extracted from different auscultation sites.

To further understand the discriminative power of these features, statistical tests such as the Mann-Whitney U test can be applied to compare the distributions of each feature between normal and abnormal groups. Significant differences in these features would confirm their relevance for classification purposes.

Incorporating these coupling features into machine learning models is expected to enhance classification performance. The multi-dimensional nature of the features allows the models to learn complex patterns associated with pathological conditions, potentially leading to more accurate and reliable diagnostic tools.

## Results

**Fig 9:**
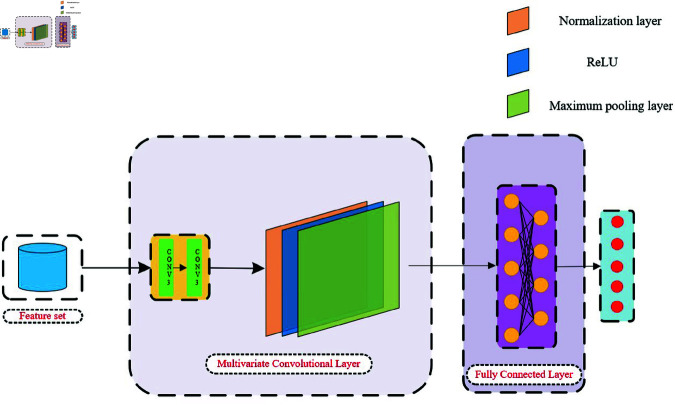
Structure of the convolutional neural network.

Traditional one-dimensional convolutional layers can only perform convolution operations using fixed-size filters, which limits their ability to capture feature information at different scales. Convolutional Neural Network (CNN) enhances traditional multilayer neural networks by introducing locally connected convolutional layers and pooling layers [26]. In this study, multidimensional convolutional layers are employed to extract features at various scales. By utilizing convolution kernels of different sizes, features at multiple scales can be simultaneously perceived and utilized. Fig 9 illustrates the structure of the CNN used in this work.

The multiscale convolutional layers use convolution kernels of different sizes, including 1×1 convolution layers. Each convolutional layer of each size includes two convolutional layers, which more effectively extract the coupling features between different pathways of heart sound signals. Through parallel concatenation operations, their outputs are connected to obtain three separate feature maps, each representing features extracted by convolution kernels of specific sizes.

Normalization, the ReLU activation function, and max pooling are subsequently applied to further process the feature maps. These operations help to regularize the data, introduce nonlinearity, and reduce dimensionality. After the sequence concatenation operation, a fully connected layer maps the high-level feature representations to the final class labels. The final output layer employs the softmax function to obtain the predicted probabilities for each category, determining the class to which the heart sound sample belongs.

Compared to one-dimensional convolutional layers, the proposed model can better capture the temporal relationships between continuous diastolic murmurs across channels, as these murmurs may exhibit certain pattern changes in adjacent time steps. The experimental setup for the algorithm in this study is configured as follows: an Intel(R) Core(TM) i5-8300H CPU @ 2.3 GHz processor, 16 GB of RAM, a 64-bit operating system, and an x64-based architecture.

Multi-dimensional coupling features were extracted from the multi-channel heart sound signals in the PhysioNet 2022 Heart Sound Challenge dataset and the clinical dataset. The ReliefF algorithm [26] was applied for dimensionality reduction, reducing the 78 extracted coupling features. Finally, binary classification of normal and abnormal signals was performed using various machine learning classifiers, including logistic regression, support vector machines, decision trees, k-nearest neighbors, Bayesian classifiers, and convolutional neural networks.

To explore the advantages of 3D time-frequency domain matrix features, machine learning techniques were utilized to perform binary classification of normal and abnormal heart sounds, and multiple comparative experiments were conducted. To control experimental variables, the dataset was proportionally divided into training and testing sets, with the training set comprising 70% of the samples and the testing set comprising 30%.

To ensure the reliability of the experimental results, both the experiments and comparative experiments used the same dataset. The evaluation metrics are defined as shown in equations (17)–(20), and the final metric values are the averages over 20 experiments.

$$\text{Acc}=\frac{TP+TN}{TP+TN+FP+FN}$$ (2)

$$\text{Pr}=\frac{TP}{TP+FP}$$ (3)

$$\text{Se}=\frac{TP}{TP+FN}$$ (4)

$$\text{Sp}=\frac{TN}{FP+TN}$$ (5)

$$\text{F1 Score}=2\times \frac{\text{Precision}\times \text{Se}}{\text{Precision}+\text{Se}}$$ (6)

In these equations, the definitions include four result types: True Positive (TP), False Positive (FP), False Negative (FN), and True Negative (TN), which are used to describe the accuracy of classification results.

### Classification of normal and abnormal heart sounds in the open-source dataset

**Fig 10:**
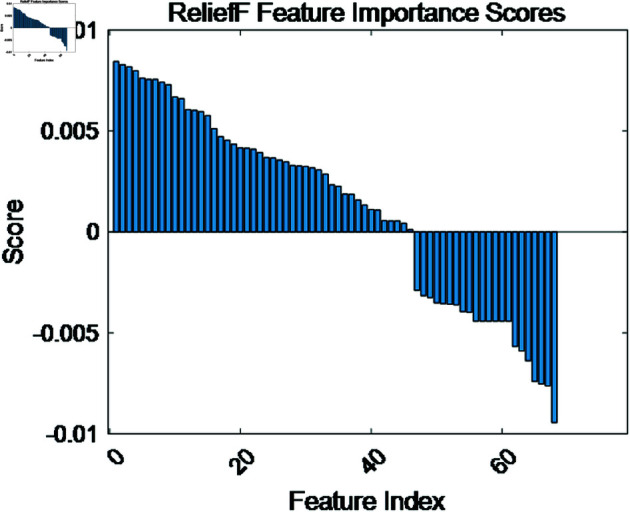
Feature importance scores for the open-source dataset.

**Fig 11:**
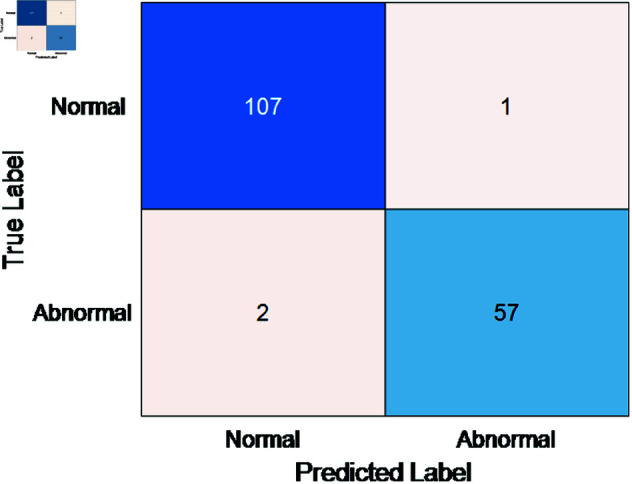
Confusion matrix for the open-source dataset.

The classification results of normal and abnormal heart sounds in the open-source dataset are presented in Table 2. It can be observed that using the coupling features reduced by the ReliefF method and the CNN classifier, the precision, sensitivity, specificity, and accuracy for the binary classification of normal and abnormal heart sounds reached 96.6%, 97.6%, 97.7%, and 98.3%, respectively. This classification performance is superior to other classifiers. Specifically, Fig 10 illustrates the feature ranking and importance scores after applying the ReliefF method, which reduced the feature dimension from 78 to 48. As shown in Table 2, the ReliefF-reduced coupling features, when used with the CNN classifier, achieved a precision of 96.6%, a sensitivity of 97.6%, a specificity of 97.7%, and an accuracy of 98.3%, demonstrating superior performance compared to other classification methods. Furthermore, under the premise of using the CNN classifier, the coupling features reduced by the ReliefF method improved the classification performance compared to the unreduced coupling features using the support vector machine (SVM). Specifically, the precision increased by 1.6%, sensitivity by 1.3%, specificity by 0.5%, and accuracy by 0.9%, and also lead to a 1.41% improvement in F1-score. Finally, the confusion matrix for the open-source dataset’s binary classification is depicted in Fig 11, which reveals only one misclassified normal and two misclassified abnormal signals. This indicates that the proposed coupling features have excellent discriminatory capability for the different signals within the dataset.

**Table 2:** Comparison of classification results before and after coupling feature dimensionality reduction under different classifiers.

Features	Classifier	Pr (%)	Se (%)	Sp (%)	Acc (%)	F1-score (%)
Unreduced Coupling Features (78 features)	Logistic Regression	90.61	91.21	91.21	91.62	90.90
	Support Vector Machine	95.00	96.40	97.20	97.40	95.69
	Decision Tree	93.40	95.80	96.30	96.30	94.58
	K-Nearest Neighbors	91.80	95.10	95.80	97.20	93.42
	Bayesian	91.90	97.20	95.40	93.50	94.48
	CNN	90.20	93.50	94.70	96.80	91.82
Coupling Features Reduced by ReliefF (48 features)	Logistic Regression	89.89	90.75	90.75	91.02	90.28
	Support Vector Machine	91.80	95.50	95.70	97.80	93.61
	Decision Tree	94.90	94.30	96.90	96.00	94.60
	K-Nearest Neighbors	91.80	95.30	95.10	96.80	93.52
	Bayesian	94.90	95.60	98.40	97.20	95.25
	CNN	**96.60**	**97.60**	**97.70**	**98.30**	**97.10**

Table notes: Comparison of different classifiers before and after feature dimensionality reduction using ReliefF. Metrics include Precision (Pr), Sensitivity (Se), Specificity (Sp), Accuracy (Acc), and F1-score (F1).

### Classification of congenital heart disease in the clinical dataset

The classification results of congenital heart disease using the reduced coupling features in the clinical dataset are shown in Table 3. It can be seen that using the coupling features reduced by the ReliefF method and the CNN classifier, the precision, sensitivity, specificity, and accuracy for classifying four types of congenital heart disease heart sounds and normal heart sounds reached 97.4%, 94.7%, 94.4%, and 95.6%, respectively. This classification performance is superior to other classifiers.

**Table 3:** Comparison of classification results before and after coupling feature dimensionality reduction under different classifiers.

Features	Classifier	Pr (%)	Se (%)	Sp (%)	Acc (%)	F1-score (%)
Unreduced Coupling Features (130 features)	Logistic Regression	87.15	81.96	81.96	91.27	83.98
	Support Vector Machine	96.94	93.20	93.50	94.40	95.03
	Decision Tree	95.82	91.20	91.20	92.40	93.45
	K-Nearest Neighbors	95.87	88.30	91.60	93.40	91.93
	Bayesian	95.90	93.10	91.20	92.70	94.48
	CNN	97.40	94.70	94.40	94.30	96.03
Reduced Coupling Features (74 features)	Logistic Regression	87.30	81.94	81.94	92.06	84.31
	Support Vector Machine	97.40	93.60	94.60	95.20	95.46
	Decision Tree	96.05	90.20	91.80	93.00	94.81
	K-Nearest Neighbors	96.37	88.90	92.60	93.50	92.48
	Bayesian	95.92	93.50	91.20	93.80	94.69
	CNN	97.40	94.70	94.40	95.60	96.03

**Fig 12:**
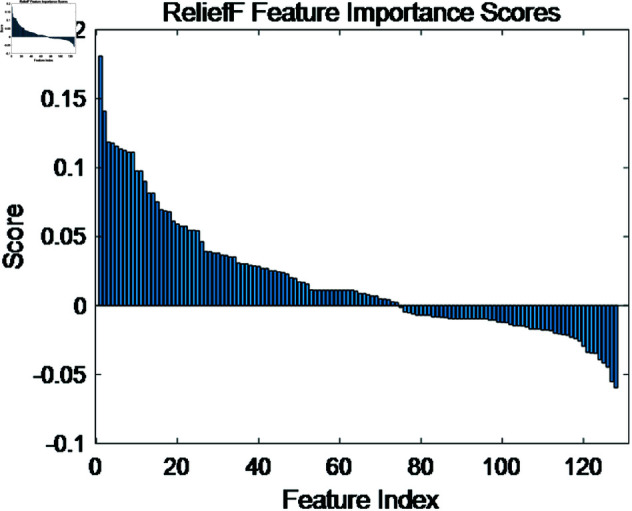
Feature importance scores for the clinical dataset.

**Fig 13:**
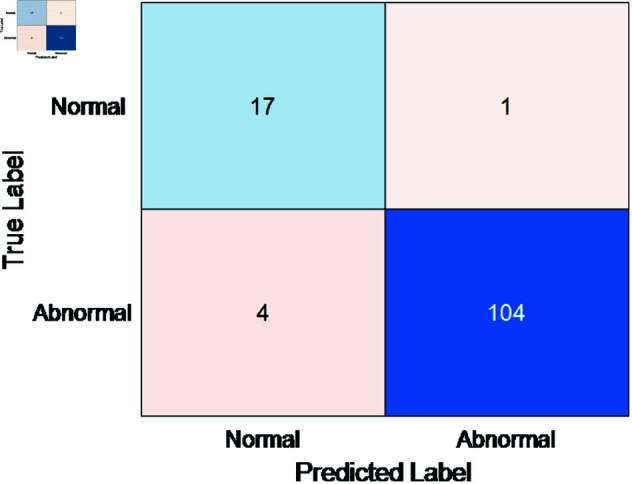
Confusion matrix for the clinical dataset.

Fig 12 displays the feature ranking and importance scores after applying the ReliefF method to the clinical dataset, reducing the feature dimension from 130 to 74. As shown in Table 3, the ReliefF-reduced coupling features, when used with a CNN classifier, achieved a precision of 97.4%, a sensitivity of 94.7%, a specificity of 94.4%, an accuracy of 95.6%, and a F1-score of 96.03%, outperforming other classification methods. Furthermore, under the premise of using the CNN classifier, the coupling features reduced by the ReliefF method improved the classification performance compared to the unreduced coupling features using the SVM classifier. Specifically, the precision increased by 0.46%, sensitivity by 0.4%, specificity by 0.9%, and accuracy by 1.3%, and also lead to a 1.0% increase in F1-score. The confusion matrix for the clinical dataset’s binary classification of normal and congenital heart disease signals is shown in Fig 13, which further validates the importance of the proposed multi-channel coupling features for CHD classification, as well as the ability of the proposed algorithm to classify different signal types within the dataset.

## Discussion

The multi-channel heart sound coupling features capture time-domain, frequency-domain, and inter-channel variations in heart sound signals, providing richer representations for classification algorithms, thereby improving classification accuracy. In this section, comparative analyses are presented in Table 4.

**Table 4:** Comparison of classification results between the proposed method and methods from the literature.

Dataset	Research Method	Pr (%)	Se (%)	Sp (%)	Acc (%)	F1-score (%)
Open-source Dataset	MFCC-KNN[14]	76.86	78.22	74.22	76.31	75.00
	Homomorphic, Hilbert, PSD, Wavelet Envelope-BiLSTM[13]	—	82.7	80.1	75.1	—
	Bispectrogram-ViT[12]	85.71	92.31	90.36	91.0	88.89
	MFCC-CRNN[7]	96.4	94.8	92.3	97.2	95.59
	Hilbert Envelope, Acoustic Features-RF[6]	96.0	95.7	93.5	94.6	95.85
	**Coupling Features-CNN**	**96.6**	**97.6**	**97.7**	**98.3**	**97.10**
Clinical Dataset	MFCC-CRNN[7]	96.4	93.6	94.4	95.2	94.98
	Hilbert Envelope, Acoustic Features-RF[6]	96.1	90.2	91.8	93.0	93.06
	**Coupling Features-CNN**	**97.4**	**94.7**	**94.4**	**95.6**	**96.03**

On the open-source dataset, the MFCC features combined with KNN, as explored in [14], resulted in precision, sensitivity, specificity, and accuracy all below 80%, indicating poor distinction between normal and abnormal heart sounds. Although the method by [13], which extracts homomorphic, Hilbert, and other features combined with a BiLSTM network, improved sensitivity and specificity by 4.48% and 5.88%, respectively, compared to [14], the overall accuracy of only 75.1% still fell short of effective signal distinction. While [12] achieved significantly improved results with precision, sensitivity, specificity, accuracy, and F1-score at 85.71%, 92.31%, 90.36%, 91.0%, and 88.89%, respectively, using a bispectrogram-ViT architecture, the computational complexity of ViT remains a concern, and its performance is contingent upon a large training dataset. Following this, the methods from [7] and [6] were applied to the open-source dataset for binary classification of normal and abnormal heart sounds, achieving accuracies of 97.2% and 94.6%, respectively, showing improved performance.

The proposed coupling features, combined with a CNN algorithm, demonstrated superior performance, reaching 96.6% precision, 97.6% sensitivity, 97.7% specificity, 98.3% accuracy, and a 97.10% F1-score. Compared to the referenced algorithms, this represents a notable improvement, with an increase of 0.2%, 1.9%, 4.2%, 1.1%, and 1.51% for precision, sensitivity, specificity, accuracy, and F1-score, respectively. Additionally, the proposed algorithm was validated on a clinical dataset. Using the coupling features with CNN, the precision, sensitivity, specificity, and accuracy were 97.4%, 94.7%, 94.4%, and 95.6%, respectively. Compared to [7] and [6], the proposed method improved precision, sensitivity, and accuracy by 1.0%, 1.1%, and 0.4%, respectively, demonstrating its effectiveness and generalizability in distinguishing between normal and congenital heart disease signals.

## Conclusion

This paper proposes a method that combines multi-channel coupling features with machine learning techniques. Using a CNN, the classification of normal and abnormal heart sounds from the PhysioNet 2022 Heart Sound Challenge dataset achieved precision, sensitivity, specificity, and accuracy of 96.6%, 97.6%, 97.7%, and 98.3%, respectively. For the classification of congenital heart disease in the clinical dataset, the precision, sensitivity, specificity, and accuracy were 97.4%, 94.7%, 94.4%, and 95.6%, respectively. The algorithm presented in this paper provides a novel and effective method for heart sound signal classification and offers significant clinical value for the identification of heart diseases. Future work will build upon the current research, which considers both time-domain and frequency-domain characteristics of heart sounds, by expanding the collection of case data for different heart disease categories. This will facilitate the development of more accurate diagnostic models for various cardiac conditions and enable a deeper understanding of the complex relationship between heart sound signals and heart health. Furthermore, exploration of additional neural network techniques to enhance model performance is planned, with the ultimate goal of achieving a real-time clinical heart sound analysis system.
